# Interval analysis of interictal EEG: pathology of the alpha rhythm in focal epilepsy

**DOI:** 10.1038/srep16230

**Published:** 2015-11-10

**Authors:** Jan Pyrzowski, Mariusz Siemiński, Anna Sarnowska, Joanna Jedrzejczak, Walenty M. Nyka

**Affiliations:** 1 Department of Adult Neurology, Medical University of Gdansk, Poland; 2 Department of Neurology and Epileptology, Medical Centre for Postgraduate Education, Warsaw, Poland

## Abstract

The contemporary use of interictal scalp electroencephalography (EEG) in the context of focal epilepsy workup relies on the visual identification of interictal epileptiform discharges. The high-specificity performance of this marker comes, however, at a cost of only moderate sensitivity. Zero-crossing interval analysis is an alternative to Fourier analysis for the assessment of the rhythmic component of EEG signals. We applied this method to standard EEG recordings of 78 patients divided into 4 subgroups: temporal lobe epilepsy (TLE), frontal lobe epilepsy (FLE), psychogenic nonepileptic seizures (PNES) and nonepileptic patients with headache. Interval-analysis based markers were capable of effectively discriminating patients with epilepsy from those in control subgroups (AUC~0.8) with diagnostic sensitivity potentially exceeding that of visual analysis. The identified putative epilepsy-specific markers were sensitive to the properties of the alpha rhythm and displayed weak or non-significant dependences on the number of antiepileptic drugs (AEDs) taken by the patients. Significant AED-related effects were concentrated in the theta interval range and an associated marker allowed for identification of patients on AED polytherapy (AUC~0.9). Interval analysis may thus, in perspective, increase the diagnostic yield of interictal scalp EEG. Our findings point to the possible existence of alpha rhythm abnormalities in patients with epilepsy.

Despite decades of experience, contemporary clinical EEG analysis remains largely focused on the identification of interictal epileptiform discharges (IEDs) - a fundamental high-specificity biomarker of epilepsy^1^. The lack of an alternative high-sensitivity marker results in an inability to effectively rule out a seizure disorder even with prolonged observation times^2^. The adult scalp EEG is, however, also abundant in more or less organized rhythmic activity – the assessment of which seems to have moved out of focus of modern electroencephalography. In this work we demonstrate the possibility to obtain new information of potential clinical value from automated analysis of the EEG alpha rhythm.

Since the advent of electroencephalography, changes in the alpha rhythm have been associated with epilepsy^3^. The degree of alpha rhythm abnormalities ranges from its complete absence^4^ to more subtle manifestations, often described as “alpha rhythm irregularity”. It has been pointed out, however, that such assessment of the alpha rhythm by visual analysis is prone to over-interpretation^5^. More recently a series of automated, computer-based methods have been tested for assessment of background EEG activity^6^,^7^, and have shown slowing of the alpha rhythm in patients with epilepsy. However, as it has been suggested, this effect could also have been attributed to the usage of AEDs^8^, the effects of which had not been controlled in these studies. Fourier analysis has been applied by Miyauchi *et al.*^9^ to demonstrate significant decrease in an alpha subband in a relatively large group of patients with epilepsy as compared with non-epileptic controls and significant increase in slow activity which was also present in a sub-group of untreated patients with epilepsy. There have been a few attempts to construct a complete computer-assisted diagnosis (CAD) tool for epilepsy based on the analysis of the interictal scalp EEG both in adult^10^,^11^ and paediatric populations^12^ – providing some optimistic results despite their complex methodologies (including the use of artificial neural networks, ANN).

EEG interval analysis was originally introduced as a computationally inexpensive alternative to Fourier spectral analysis^13^. Lacking the convenience of being a linear transformation it had not, however, gained much popularity, especially as the power of available computers continued to increase and the Fast Fourier Transform (FFT) was introduced in the 60’s^14^. The formal mathematical relationship linking interval analysis to Fourier analysis was established by Kedem *et al.*^15^. Despite its simple definition, interval analysis has complex mathematical properties and is closely related to nonlinear signal analysis methods^16^. It has recently emerged once again as a useful tool for data compression in nonlinear signal analysis and was successfully applied to human scalp EEG data^17^,^18^. We demonstrate that this partially forgotten tool, when used for alpha rhythm assessment, provides putative new epilepsy-specific markers which may, in perspective, be used to improve the diagnostic yield of standard scalp EEG recordings.

## Methods

We retrospectively studied EEG recordings from 78 patients (20 male and 58 female, aged 18–68 years, mean 35 years) who were hospitalized in the Departament of Neurology and Epileptology, Medical Centre for Postgraduate Education, Warsaw, Poland during the years 2009–2013. All data analysed were collected as part of routine clinical workup. The protocol of the study was approved by the Medical University of Gdańsk Independent Bioethics Commission for Research and all analysis was performed in accordance with the approved guidelines. The presented data do not contain any identifying information relating to the patients. All data analysis was performed in MATLAB (Mathworks Inc., 2012).

### Characteristics of the studied group

The patients were selected due to the definiteness of their diagnoses. Within the studied group 51 of the subjects were diagnosed with epilepsy (19 with TLE and 32 with FLE). A proportion of these patients were hospitalized because of the resistance to antiepileptic drugs but available documentation did not include complete medication history in some of the remaining subjects. The duration of epilepsy (time since the occurrence of their first seizure) was known in 44 subjects (median 17 years, range 1–51 years) and, since patients with epilepsy were not seizure free during the time preceding hospitalization, it was taken as an indirect measure of the degree of drug resistance. The control group comprised two subgroups: 13 patients with PNES and 14 patients who were admitted to hospital because of headache (of whom none were diagnosed with epilepsy or PNES). In all cases of PNES diagnosis was confirmed by video-EEG monitoring. Additional information on individual patients in the studied group is provided in Supplementary Table 1.

N = 60 (77%) of the patients were receiving AEDs at the time of examination (24, 21 and 15 were on one, two and three AEDs respectively). The heterogeneity of drugs taken by the patients did not allow to distinguish single-AED effects. The potential impact of AEDs on the patients’ EEG was therefore assumed to be proportional to the number of different AEDs taken by a subject (nAED, ranging from 0 to 3) regardless of individual drug dosing. Benzodiazepines with mainly sedative-hypnotic properties were also included in nAED due to their considerable influence on background EEG activity. Diagnosis and nAED were significantly correlated - i.e. patients with the diagnosis of epilepsy were more likely to be on AED therapy (nAED > 0, P < 0.001, chi^2^ test) and patients given more AEDs were more likely to be diagnosed with epilepsy (P < 0.001 for nAED > 1 and P = 0.0017 for nAED > 2, chi^2^ tests). AEDs were, however, also taken by the majority of the PNES patients (85%) and this control subgroup did not differ from the epilepsy group in this respect (P = 0.405, chi^2^ test).

### Recording procedures and the EEG-score

Scalp EEGs were recorded according to a standard 20-minute clinical protocol including hyperventilation (Hv) and photic stimulation (Fs) using the same recording system for all patients (ELMIKO Medical). The average duration of recording was 20.2 minutes, range 19.5–22.1 minutes). Recording electrodes were placed according to the international 10–20 system and reference electrodes were placed on the patients’ nape, at the level of vertebra prominens. The signal was passed through an analogue 0.5Hz high-pass filter and digitized at 250 Hz. Data from the standard 19 scalp channels in referential montage were then analysed mathematically. The review summaries of EEG recordings were converted to a 4-level scale of pathology (the “EEG-score”): (0) no abnormalities, (1) normal EEG variants, (2) non epilepsy-specific abnormalities (including background slowing) and (3) epilepsy-specific abnormalities (IEDs). No studied recording contained any overt ictal electrographic activity.

### Signal pre-processing and zero-crossing interval analysis

The signals from individual channels were first digitally band-passed using zero-phase shift FIR filters. The filter functions were constructed using the “fir2” Matlab function. The passband frequencies were fixed at theta-alpha [4–13 Hz] for initial statistical analysis and treated as a free parameters (in 1 Hz increments) in classifier fitting for cross-validation (see below). The frequency response of a typical filter is shown in Supplementary Fig. 1.

Recording segments were defined according to the EEG recording protocol (pre-Fs, Fs, post-Fs, Hv and post-Hv). Sub-segments of the post-Hv segment were also analysed (see Results). Eyes-open and eyes-closed conditions were not considered separately as this did not substantially influence the results (data not shown). Interval histograms obtained from different EEG channels were analysed as sums of symmetric channels (e.g. Fp1+Fp2, T3+T4 etc.) which allowed to reveal spatial effects regardless of the lateralization of epileptic foci. Pooling of all segments and channels together is referred to as “whole recording” analysis throughout the following text.

Zero (i.e. isoelectric line) crossings of the pre-processed EEG signal were identified by linear interpolation (numerical differentiation of the signal and shifting the threshold used for crossing detection did not substantially influence the results - data not shown). The distributions of the relative counts of time intervals between subsequent zero-crossings (the “interval spectrum”) were estimated by histogramming (using bins of 4-ms width, range 0–4000 ms, variation of the histogram bin width did not affect the results in a qualitative manner - data not shown). This was followed by pooling of raw histograms over channels and/or segments (see above) and histogram normalization.

The family of filters used for prefiltering was kept fixed. Hence the algorithm transforming the EEG signal to its interval spectrum had 4 free parameters: the lower passband (1), the upper passband (2) together with the choice of recording segments (3) and channels (4) to be included in the pooled interval histogram. In case of whole recording analysis only the passbands (1 and 2) needed to be specified. The power spectral densities of (prefiltered) signals were additionally estimated using Welch’s method (4s-window).

### Statistical analysis

We assessed three types of interval spectrum-derived markers: (1) the relative counts of fixed-length intervals (e.g. the counts of 100ms intervals were tested as a putative marker - this approach may be viewed as a form of “spectrometry”), (2) the standard descriptive statistical parameters of the interval spectrum (mean, mode, median, standard deviation, interquartile range) and (3) selected entropy measures of the interval spectrum (Shannon entropy Hs^19^ and min-entropy H_min_) which quantify the level of disorder associated with a probability distribution. The entropy functions are defined as follows:


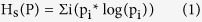



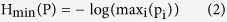


where P denotes the normalized interval spectrum (a discrete probability distribution), p_i_ is the relative probability of finding an interval in the i-th bin, and max_i_ denotes the maximum over all bins. Hs and H_min_ are special cases of a more general entropy measure^20^. H_min_ is a limiting case sensitive only to the area of the highest probability accumulation and reduces to the negative logarithm of the highest distribution peak. Entropy measures have been used previously in EEG analysis in the context of automated seizure detection^21^,^22^.

The distributions of analysed measures within patient subgroups did not pass normality and homoscedasticity tests. Therefore, in order to avoid violation of ANOVA assumptions, we tested the data using nonparametric one-way Kruskal-Wallis ANOVA separately for the effects of diagnosis (4 groups) and nAED (4 groups). Multiple statistical hypothesis testing has been accounted for by using Bonferroni’s correction. Post-hoc multiple comparisons were performed following Tukey’s procedure. In order to focus on robust candidate markers and reduce the number of statistical tests performed, we analysed only histogram bins for which the raw interval count was >100 for all patients.

### ROC analysis and cross-validation

Discriminatory power of identified putative markers was assessed in the framework of receiver-operating-characteristic (ROC) analysis. Epilepsy (FLE and TLE) and control (headache and PNES) subgroups were joined to enable binary classification. The area under the ROC curve (AUC) was used as a measure of classifier performance. As a summary statistic AUC is equivalent to the probability that the score attributed to a randomly chosen case is higher than that attributed to a randomly chosen control. When calculated “naively” for the whole patient group AUC is, however, prone to optimistic bias due to overfitting^23^ and should not be considered a reliable predictor of real-world performance. To overcome this issue we used an approach called leave-pair-out cross validation^23^,^24^, in which a case-control pair is omitted from the data set and the parameters of the algorithm are fitted to maximize AUC for the remaining patient group. The scores for the omitted pair are then calculated according to the fitted algorithm and the procedure is repeated for all possible case-control pairs (“folds”). AUC is estimated by the fraction of case control-pairs for which the case scores are higher than the control. AUC values obtained in this way are denoted by AUC* and AUC** throughout the text depending on whether the algorithm was fitted to whole-recording interval spectra (“one-step fitting”) or to individual channels and segments (“two-step fitting”, see Results). A potentially useful marker was assumed to be associated with AUC*/** greater than 0.75 (or equivalently less than 0.25, as AUC transforms to 1 - AUC under the “inversion” of the classifier - the exchange of “positive” and “negative” groups).

## Results

### Initial statistical analysis - screening for putative markers

As our primary interest was to study the alpha rhythm and its possible slowing, initial statistical analysis was performed for signals band-passed in the theta-alpha [4–13 Hz] band. Markers significantly influenced by either the patients’ diagnosis or nAED were further evaluated using ROC analysis with cross-validation (see the following section).

**Figure 1:**
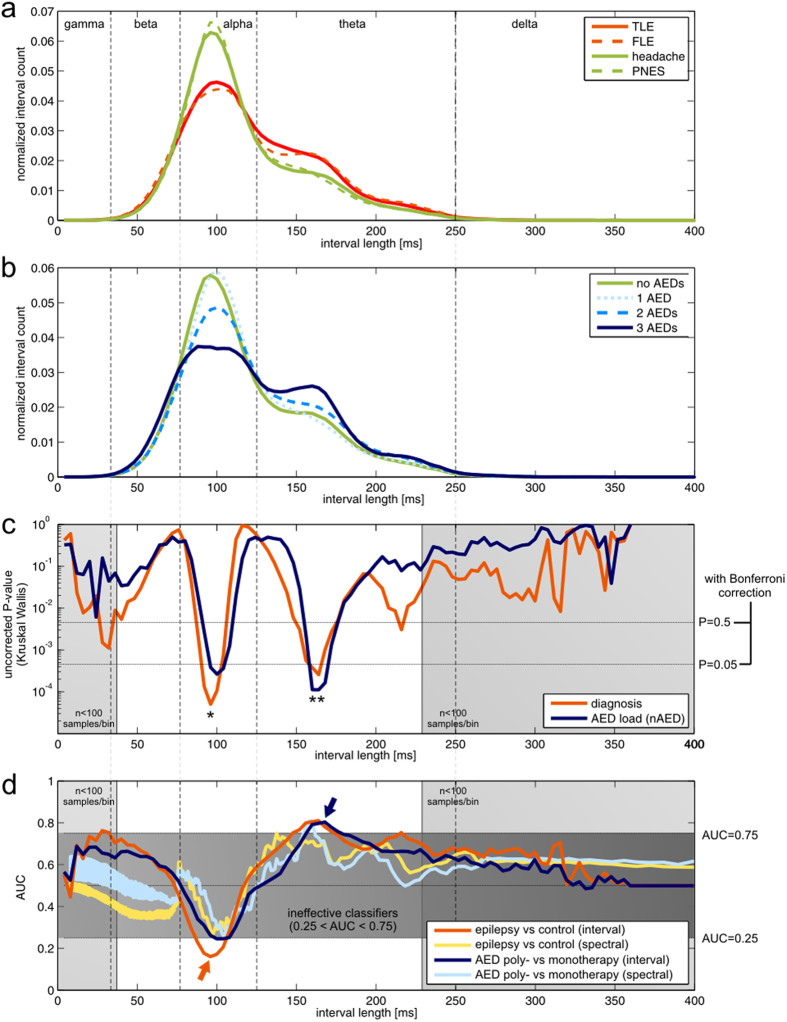
Statistical analysis of zero-crossing interval spectra (whole recordings). The vertical lines demark interval length ranges corresponding to commonly used electroencephalographic frequency bands. Note that increased counts of intervals of a certain length do not necessarily correspond to a sinusoidal rhythm and so, even though oscillation frequency and period are inversely related, Fourier spectral bands cannot be viewed as a simple equivalent of corresponding interval ranges (e.g. the 8–13 Hz alpha band and the respective 77–125 ms alpha range). (**a**) Averaged interval spectra for different diagnosis subgroups. The peak in the alpha interval range is visibly lower in epilepsy subgroups (red lines) than in control patient subgroups (green lines). Increased contribution of theta intervals to the spectrum is also seen in patients with epilepsy. (**b**) Averaged interval spectra for subgroups with different nAED. Changes qualitatively similar as described above are associated with increasing number of AEDs taken by the patients (blue lines). (**c**) Statistical analysis performed for fixed interval lengths: P-value vs interval length relationships for the effects of diagnosis (red line) and of nAED (dark blue line). Horizontal lines represent Bonferroni-corrected P value levels. The significant diagnosis effect dominates in the alpha-range (single asterisk) while the theta-range contains comparable overlapping significant effects of both independent variables (double asterisk, the nAED effect is more significant). Light grey areas represent interval ranges that were rejected form analysis (see Methods) (**d**) “Naive” ROC analysis performed for fixed interval lengths. AUC vs interval length relationships are shown for epilepsy vs control and AED polytherapy vs monotherapy classification problems (see also Suppl. Fig. 2). The dark-grey area represents no potentially effective classification (0.25 < AUC < 0.75). For interval spectrum based classifiers (red and dark blue lines) arrows mark the curves’ extremal points which correspond to potentially useful markers. AUC-inverse frequency relationships obtained for a Fourier power spectrum based classifiers (yellow and light blue lines) are shown for comparison. Trends for the decrease in alpha and increase in theta are consistent across both methods.

The shapes of averaged interval spectra (obtained from whole recordings) for different patient subgroups are shown in Fig. 1a. From their general inspection one can see that patients with epilepsy have reduced counts of intervals within the alpha range with concomitantly increased interval counts in the theta range. Differences between epilepsy (FLE vs TLE) and control (PNES vs headache) subgroups are much less pronounced. Qualitatively similar changes are, however, associated with AED load (nAED) in a “dose-dependent” manner (Fig. 1b) – patients receiving more AEDs have decreased alpha and increased theta interval counts. As the two independent variables (diagnosis and nAED) were significantly correlated (see Methods), it was vital to distinguish between their individual effects.

Simultaneous one-way ANOVA (n = 78 for all tests, number of tests for Bonferroni correction = 110) showed that significant effects lie in two disjoint portions of the interval spectrum (Fig. 1c). Within the alpha range interval counts were strongly influenced by the patients’ diagnosis (peak P = 0.0055 for intervals of length = 96ms - Fig. 1c, single asterisk). This effect was, however, confounded by a less significant effect of nAED (peak P = 0.0290 for intervals of length = 100ms). Multiple comparisons confirmed significant reduction of alpha interval counts in epilepsy as opposed to control subgroups without significant differences between epilepsy (FLE vs TLE) and control (headache vs PNES) subgroups (data not shown). Increased nAED, on the other hand, was associated mainly with an increase in theta-range interval counts (peak P = 0.0123 for interval length = 164ms, Fig. 1c, double asterisk). This effect was confounded by a significant overlapping effect of diagnosis (peak P = 0.0282 for the same interval length). Multiple comparisons revealed significant differences only between monotherapy and polytherapy subgroups (data not shown).

P-values associated with descriptive and entropy measures are summarized in Table 1. Both entropy measures differed significantly between diagnosis groups (P = 0.0480 and P = 0.0027 for H_S_ and H_min_ respectively) with no significant effects of nAED (P > 0.5 and P = 0.09217 respectively, see Table 1). As in the case of alpha-range intervals, multiple comparisons showed that increased entropy values differentiated epilepsy from control subgroups (data not shown). A trend towards increase of median interval values was found in the epilepsy group (P = 0.0604) which may point to background rhythm slowing in these patients.

### “Naive” ROC analysis

Figure 1d shows AUC-interval length relationship curves for two main classification problems (epilepsy vs control and AED polytherapy vs monotherapy). Here AUC values have been calculated “naively” for the whole patient group as a summary statistic (i.e. the probability of ranking cases higher than controls). The shape of these curves was relatively stable across different filtering bands - with a characteristic minimum in the alpha range followed by a maximum in the theta range (arrows). The position of these extremal points was used in classifier optimization (see next section). Accordingly and in line with the above findings, AUC values >0.75 or <0.25 were found only in alpha and theta interval ranges. Fourier power density amplitudes were analysed in the same manner as interval spectra and the resultant AUC-inverse frequency relationship curves are shown for comparison. It can be seen that the trends towards alpha reduction and theta increase are consistent across both classifier families even though AUC values in the alpha range are markedly lower for interval spectrum based classifiers. “Naive” AUC also exceeded 0.75 for both classifiers based on the entropies of the interval spectrum (data not shown).

Analogous “AUC curves” for additional classification problems (TLE vs FLE, PNES vs headache, epilepsy duration > median vs < median and on-AED vs off-AED) are presented in Supplementary Fig. 2. For all interval lengths 0.25 < AUC < 0.75 which suggests that interval spectrum magnitude-based markers are not suitable for effective classification. The same holds true for descriptive statistical and entropy measures (data not shown).

Interestingly, despite the fact that the signals were initially band-passed in the theta-alpha band, a small number of beta and gamma range intervals were detected – particularly in patients with epilepsy (this potentially relevant effect is visible in Fig. 1c,d, left shaded areas). We did not further consider these portions of the interval spectrum because of insufficient interval statistics (see Methods). The nature of this “interval leakage” phenomenon and its possible relationship with the presence of IEDs in the recordings requires further study. Of note, such leakage seems not to be present for spectral classifiers (AUC trends become inconsistent between spectral and interval classifiers in the beta and gamma range - see Fig. 1d).

### ROC analysis with cross-validation

Basing on the results obtained in the previous section, four potential interval spectrum-derived markers were conceptualized. Discrimination of patients with epilepsy form controls was assessed for: (1) the “alpha score” - a classifier based on epilepsy-related reduction of interval spectrum magnitude in the alpha range and (2,3) two “entropy scores” - basing on increased entropy of the interval spectrum in patients with epilepsy (both entropy measures were analysed for this purpose). Discrimination of AED polytherapy from monotherapy patients was assessed for (4) a classifier based on increased interval spectrum magnitude in the theta range - the “theta score”. Discriminative power (AUC* and AUC** for one- and two-step optimization respectively) was estimated by cross validation (see Methods) and the results are summarized in Table 2. ANOVA testing was repeated for the identified optimal band-pass filter settings to confirm the statistical properties of the studied markers. Fourier power spectrum equivalents of the alpha- and theta-score were also analysed for the purpose of comparison (Fourier amplitudes were treated analogously to those of interval spectra).

### One-step optimization - analysis of whole recordings

The alpha-score classifier was optimized by minimizing AUC over the alpha interval range (i.e. finding the minimum of the AUC-interval length curve - see Fig. 1d, red arrow) and over different combinations of lower and upper passband frequencies followed by inverting the classifier’s output. Cross validation revealed effective discrimination of patients with epilepsy from controls (AUC* = 0.8134). Optimal classification was most commonly associated with the [3–13 Hz] passband and interval length = 96 ms. Repeated statistical analysis for these parameter values revealed, however, the persistence of a confounding nAED-related effect (P = 0.0318, see Table 2). The Fourier equivalent of the alpha-score did not show sufficient discriminatory power (AUC* < 0.75).

Entropy-score classifiers were optimized by maximizing AUC over different combinations of passband frequencies. Cross-validated AUC* exceeded 0.75 only for the Shannon entropy-score (AUC* = 0.7640) and this classifier benefited from relatively narrow-band pre-filtering [7–13 Hz]. As for initial statistical analysis (see previous section) no significant dependence of either of entropy scores on nAED was found (P = 0.0770 and P = 0.3696 for Shannon- and min-entropy scores respectively).

The theta-score classifier was optimized by maximizing AUC over the theta interval range (i.e. finding the theta-range maximum of the AUC-interval length curve - Fig. 1d, blue arrow) and different combinations of lower and upper passband frequencies. Cross-validated AUC* = 0.9063 and was associated with wide-band [0–15 Hz] filtering and interval length of 176ms. Repeated statistical analysis revealed a relatively weak confounding effect of diagnosis (P = 0.0218). The Fourier equivalent of the theta-score was less efficient but also able to discriminate AED polytherapy from monotherapy patients (AUC* = 0.7917).

Both alpha-score and Shannon entropy-score classifiers retained their discriminative power when analysis was restricted to patients with normal or normal-variant EEGs (EEG score = 0 or 1) with AUC* = 0.8057 and 0.7829 respectively (see Table 2, last column). The theta-score classifier’s performance was, however, markedly reduced (AUC* = 0.6381), possibly owing to the exclusion of patients with overt background slowing (EEG score = 2).

The distributions of average AUC values obtained during classifier fitting are shown as a function of passband frequencies in Supplementary Fig. 3. Of note, high AUC values were obtained only when the alpha band was contained in the assessed passband.

### Fitting over channels and segments

**Figure 2:**
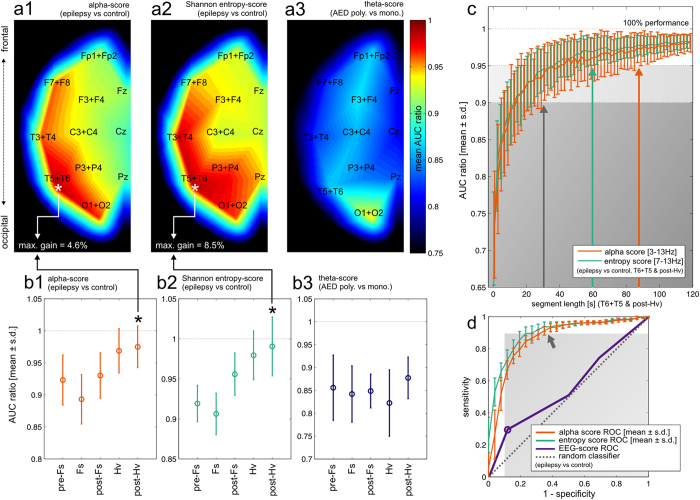
Trends in the optimization of alpha-, Shannon entropy- and theta-score based classifiers. Note that AUC values and ROC curves displayed in this figure serve only to illustrate trends in the process of optimisation instead of being results of cross-validation (which are summarized in Table 2). The results are displayed as AUC ratios (see Results). (**a1–a3**) The distributions of mean AUC ratios over EEG channel positions. It can be seen that alpha- and Shannon entropy-scores perform best for temporal-occipital leads (white asterisks) while the theta-score performance is uniformly reduced across individual channels with the exception of O2 + O1. (**b1–b3**) The dependence of mean AUC ratios on the recording segments. For alpha- and Shannon entropy-scores performance increases towards the end of the recording with a maximum in the post-Hv segment (black asterisks). (**c**) The expected dependence of performance (alpha- and Shannon entropy-scores) on segment length (T6 + T5 & post-Hv, 1–120 s). 100% performance refers to “naive” AUC obtained for the full-length segment. Grey shaded areas correspond to 90% and 95% performance and arrows indicate the minimum segment lengths for which respective performance levels are reached (see Results for details). (**d**) Averaged ROC curves (see Results for details) for alpha- and Shannon entropy-scores (red and green curves respectively). The ROC curve associated with the EEG-score is shown for comparison (violet curve, the circle represents classification based on the presence of IEDs). The grey shaded area corresponds to sensitivity or specificity <90%. Interval spectrum-based markers achieve >90% sensitivity for ~60–70% specificity (arrow).

The properties of identified markers were further assessed by extending the fitting procedure by a second step: after finding the optimal passband and interval length AUC was maximized over all possible channel/segment combinations (see Methods). The results are summarized in Table 2. Cross-validated values obtained from this two-step fitting procedure (AUC**) showed that additional increase in discrimination power was available only for the entropy scores (with 6.9% and 3.3% increases for the Shannon and min-entropy scores respectively). As in the previous section, the AUC values obtained during classifier fitting were kept and analysed for relevant trends (Fig. 2, Suppl. Fig. 4). The results for the min-entropy score are not displayed due to inferior efficiency of this marker.

The potential gain obtained from analysing specific channel/segment combinations instead of whole recordings is expressed by mean AUC ratios (i.e. AUC values calculated during the second fitting step normalized by corresponding maximized AUC values obtained in the first step, averaged over all cross-validation folds). For alpha- and Shannon entropy-scores AUC ratios were consistently greatest in temporal-occipital leads and towards the end of the recording (asterisks in Fig. 2a1-a2, and 2b1-b2). The most common optimal channel/segment combination was the same for both markers (T6+T5 & post-Hv) offering maximum average AUC gain of 4.6% and 8.5% respectively. Interestingly, this temporal-occipital pattern also held true when TLE patients were excluded from the analysis (data not shown).

The theta-score performance, on the other hand, did not benefit form restricting analysis to individual channels and segments. The loss of AUC was the smallest in occipital channels (~5%) and did not display any particular temporal pattern (see Fig. 2a3 and 2b3). Supplementary Fig. 4 shows the detailed distributions of mean AUC ratios for individual channel/segment combinations.

### The effect of segment length

We next analysed how the performance of alpha- and Shannon entropy-score based classifiers depended on the length of the analysed recording segment. Instead of analysing the whole T6 + T5 & post-Hv segment, a random subsegment of fixed duration was chosen independently for each patient and “naive” AUC values for alpha- and entropy-scores were computed. This procedure was repeated 100 times and the results (mean AUC ± s.d. as a function of subsegment length) were normalized by AUC obtained for the whole T6 + T5 & post-Hv segment. As shown in Fig. 2c. both markers can be expected to reach 90% performance for segment lengths >30s while segments of at least 60–90 s would be required to obtain 95% performance. The Shannon entropy-score slightly outperformed the alpha-score.

### Comparison to visual EEG analysis

The comparison of ROC curves (corresponding to maximized AUC values obtained in subsequent folds of two-step optimization, averaged over specificity values) shown in Fig. 2d shows potential superior high-sensitivity performance of the alpha- and Shannon entropy scores when compared to a classifier based on the EEG score (see Methods). Interval classifiers reach >90% sensitivity at ~60–70% specificity (arrows). Note that these ROC curves were not obtained by cross-validation and may be biased due to in-sample optimization. The expected high-specificity performance of visual EEG analysis^1^ was confirmed by ~90% specificity at ~30% sensitivity of the EEG-score classifier (circle).

## Discussion

Our results demonstrate the possibility to distinguish patients with epilepsy from non-epileptic controls by interval analysis of interictal EEG recordings. Classification in the studied patient group was possible at relatively high sensitivity and specificity (AUC~0.8). This level of performance is comparable to published ANN-based methods^10^,^11^. Statistical and ROC analysis emphasized two putative epilepsy-specific markers: the relative count of intervals in the alpha range (the “alpha-score”) and the Shannon-entropy of the interval spectrum (the “Shannon entropy-score”). When compared to IED-based visual analysis, these interval based classifiers may offer superior performance in the high-sensitivity regime.

Alpha-score and Shannon entropy-score based classifiers performed best for signals obtained from temporal and occipital leads (i.e. where the alpha rhythm is usually most prominent) and for recording segments following hyperventilation. Effective classification may be possible with only ~1 minute long recording segments. As a practical consequence, the lack of requirement for a complete set of EEG leads and for prolonged recordings may potentially lead to an applicability of the method e.g. in the emergency department or outpatient setting.

Decreases in alpha activity in patients with epilepsy as compared with controls were previously reported but were not notable in a subgroup of untreated patients^9^. Slowing of the alpha rhythm in patients with epilepsy^6^ was reproduced in our group by a trend towards increase of median interval length. The weakness of this effect may be partially accounted for by the heterogeneity of AEDs taken by our patients. The inability to distinguish patients with longstanding epilepsy form those more recently diagnosed by interval spectrum-derived markers suggests that the studied phenomenon may be characteristic not only for longstanding drug-refractory epilepsy.

Even though a proportion of patients with epilepsy were refractory to AED treatment and often on AED polytherapy, the inclusion of PNES patients (of whom many were also administered one or more AED) in the control group allowed for statistical analysis which revealed weak and non-significant AED dependence of alpha- and Shannon entropy-scores respectively. AED-related effects were concentrated in the theta interval range and allowed for identification of AED polytherapy patients in the studied group. Differing spatial patterns of optimal epilepsy vs control and theta-score based AED polytherapy vs monotherapy classification further support the distinctness of epilepsy and AED-related phenomena. The association of theta band effects with AEDs has been reported in previous studies^9^,^25^. It remains to be verified whether the theta-score could be used as an indicator of AED-related toxicity.

When interval and Fourier spectral analysis were applied analogously to the same data, consistent trends were seen in the alpha and theta bands. Interval analysis, however, outperformed Fourier analysis in the alpha range and effective classification is likely not to be possible using simple Fourier spectrum based classifiers. We hypothesize that this may, at least in part, result from different sensitivity of both methods to episodic low-frequency EEG artefacts (e.g. oculographic, movement and some electrode artefacts). In case of interval analysis, large deflections of the signal give rise to few unusually long intervals which are likely to fall outside the region of interest identified in our study (the theta-alpha range). Contrastingly, time-localized waveforms typically make a broadband contribution to Fourier power spectra and may contaminate the bands of interest. The transformation of the signal to its interval spectrum could thus naturally “filter out” recording segments contaminated with considerable low-frequency artefacts. High-frequency artefacts were, on the other hand, mostly removed by pre-filtering (see Methods). An in-depth comparison of Fourier and interval-based EEG analysis requires futher study.

The preservation of discriminative power of interval spectrum markers and the EEG score when analysis was restricted to patients with normal and normal variant EEGs suggests that epilepsy-specific EEG abnormalities captured by the interval spectrum may not be IED-related. As in the case of episodic low-frequency artefacts it may be conjectured that interval analysis is in general not sensitive to IEDs as they are not likely to produce sufficient interval counts to influence the overall statistic. It must be stressed that our findings should be considered preliminary and require validation in a larger and homogenous patient populations. Nevertheless, it seems that the information extracted from EEG recordings by interval and visual analysis may be to some extent complementary.

Some electroencephalographers make note of the qualitative features of background EEG rhythms. “Sharpened” and “irregular” alpha rhythm morphologies are often found in recordings from patients with epilepsy even in the absence of overt IEDs. While clinical reasoning based on such features is controversial and widely regarded as overinterpretation^5^, our findings support the view that alpha rhythm abnormalities may actually exist and be epilepsy-specific. The entropy-score may in particular be considered a direct measure of rhythm irregularity and its significant, AED-independent increase occurs in patients with epilepsy over cortical regions where normally the alpha rhythm is most pronounced. Interestingly, this effect is not restricted to patients with TLE which points to the possibility of diffuse brain pathology. The physiological function of the alpha rhythm is not fully understood, yet there exists a body of evidence pointing towards its inhibitory role in the cortex^26^. Alpha rhythm abnormalities might therefore be a manifestation of cortical disinhibition.

## Conclusions

We demonstrate that zero-crossing interval analysis of interictal EEG recordings may be used for the distinction of patients with focal epilepsy from non-epileptic controls including patients with PNES. With the development of this method it may become possible to augment the predictive value of the standard EEG for epilepsy work-up. The pathophysiological interpretation of our findings points to the possible existence of diffuse alpha rhythm abnormalities in patients with epilepsy.

## Additional Information

**How to cite this article**: Pyrzowski, J. *et al.* Interval analysis of interictal EEG: pathology of the alpha rhythm in focal epilepsy. *Sci. Rep.* **5**, 16230; doi: 10.1038/srep16230 (2015).

## Supplementary Material

Supplementary Figures and Tables

## Figures and Tables

**Table 1:**
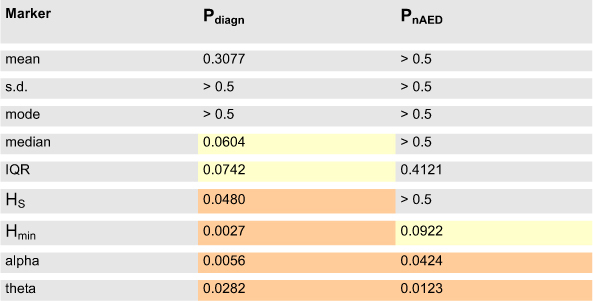
Summary of initial statistical analysis for interval spectrum based candidate markers.

Abbreviations: IQR - interquartile range, HS - Shannon entropy, Hmin - min-entropy. Pdiagn and PnAED denote Kruskall-Wallis P-values associated with the effect of diagnosis and nAED respectively. Significant P-values (using Bonferroni correction) are marked in red and trends towards significance (0.05 < P < 0.10) in yellow. As can be seen only HS and Hmin depend significantly on diagnosis and are not significantly influenced by nAED. Alpha and theta band P-values correspond to the minima marked by single and double asterisks in Fig. 1c respectively.

**Table 2:**
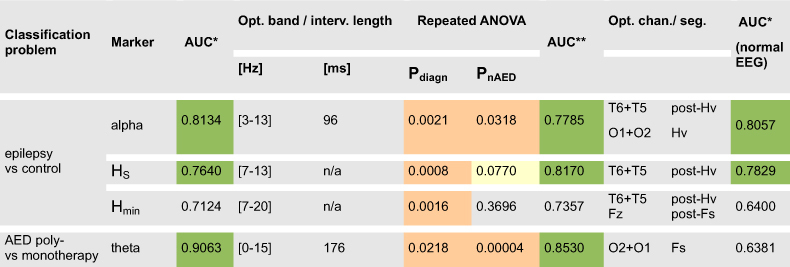
The results of ROC analysis in the framework of cross-validation.

AUC* and AUC** represent values obtained for one- and two-step optimization respectively. Significant P-values (using Bonferroni correction) and AUC values > 0.75 are marked in red and green, respectively. It can be seen that only alphascore and Shannon entropy-score based classifiers are effective in discriminating patients with epilepsy form controls under all studied conditions (one-step optimization, two-step optimization and one-step optimization restricted to normal and normal variant EEGs). Pdiagn and PnAED denote Kruskall Wallis P-values associated with the effect of diagnosis and nAED respectively and have been obtained for prefiltering and interval length values most commonly chosen in the process of classifier optimization (opt. band/interv. length). As can be seen the entropy-score classifiers are characterized by independence of AED-related effects.
