# Supplementary material for: Interval analysis of interictal EEG: pathology of the alpha rhythm in focal epilepsy
Source: Sci Rep. 2015 Nov 10;5:16230. doi: 10.1038/srep16230 (PMC4639771; doi:10.1038/srep16230)
Supplement: Supplementary Figures and Tables [file srep16230-s1.pdf]

Interval analysis of interictal EEG: pathology of the alpha rhythm in focal epilepsy.

Jan Pyrzowski, Mariusz Siemiński, Anna Sarnowska, Joanna Jedrzejczak, Walenty M. Nyka

Supplementary Information

Supplementary Figure 1.

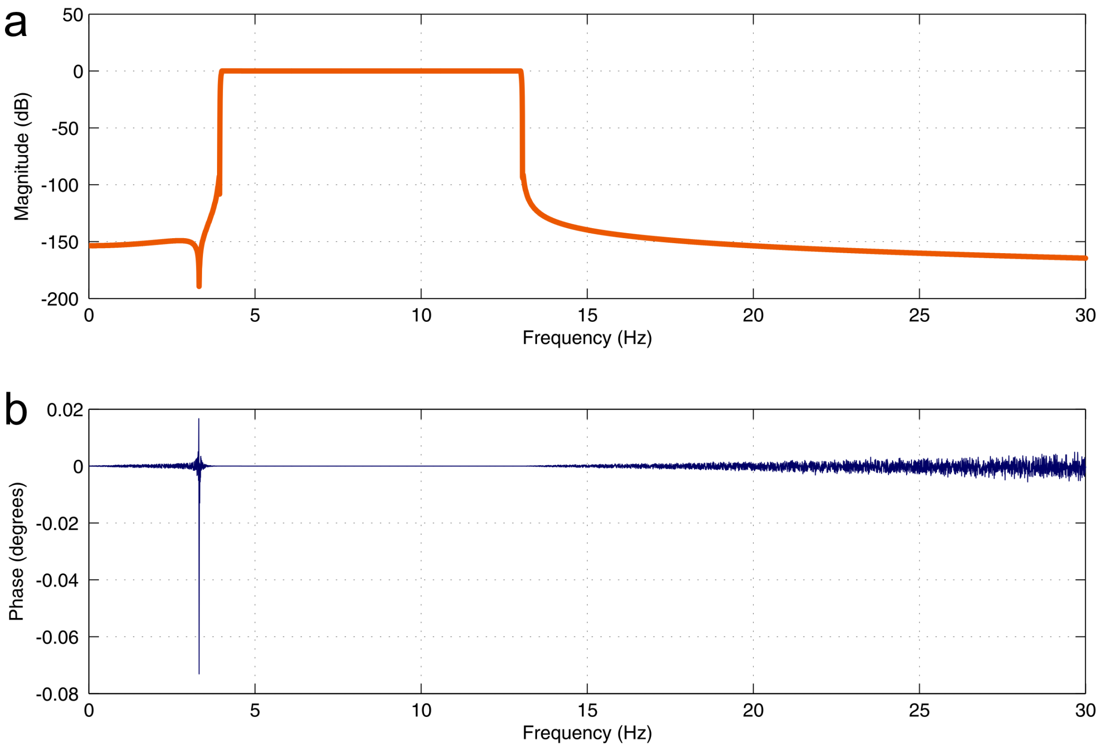

Frequency response of a representative filter ([4-13Hz] band-pass) used in signal preprocessing (see Methods). (a) Magnitude response. (b) Phase response.

Supplementary Figure 2.

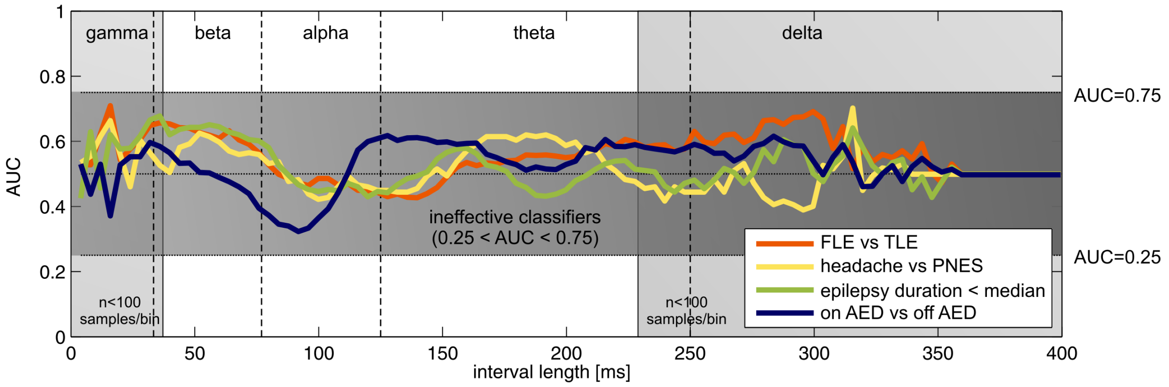

“Naive” AUC-interval length relationship curves for additional classification problems (supplement to Fig. 1d): discrimination between diagnosis subgroups (FLE vs TLE, headache vs PNES), discrimination of patients with epilepsy basing on the duration of disease (>median duration vs < median duration) and discrimination of patients on AED from those not taking medication. For all curves 0.25<AUC<0.75 which points to no potentially effective interval spectrum magnitude based classification.

Supplementary Figure 3.

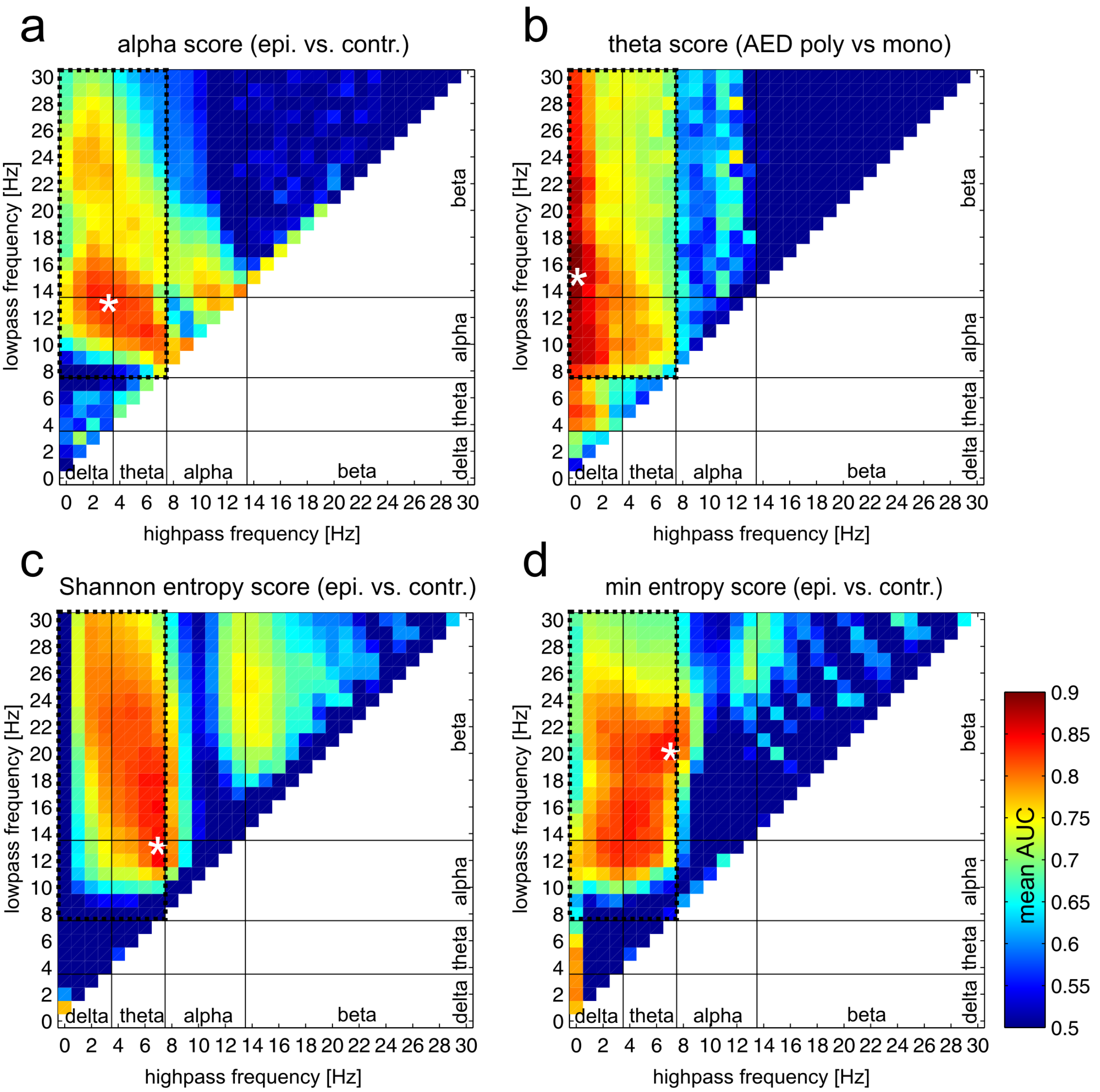

Trends in the optimization of alpha-, entropy- and theta-score based classifiers with respect to prefiltering passbands (one-step fitting - see Results). The colour scale is common for all 4 panels and represents AUC values obtained during classifier fitting and averaged over all cross-validation folds (for the results of cross-validation - see Table 2). As can be seen AUC varies smoothly across wide regions of passband values. AUC>0.75 occurred almost exclusively when the passband included the alpha band (regions demarked by dashed lines). Asterisks represent most common passband combinations that maximized classifier performance (see also Table 2).

Supplementary Figure 4.

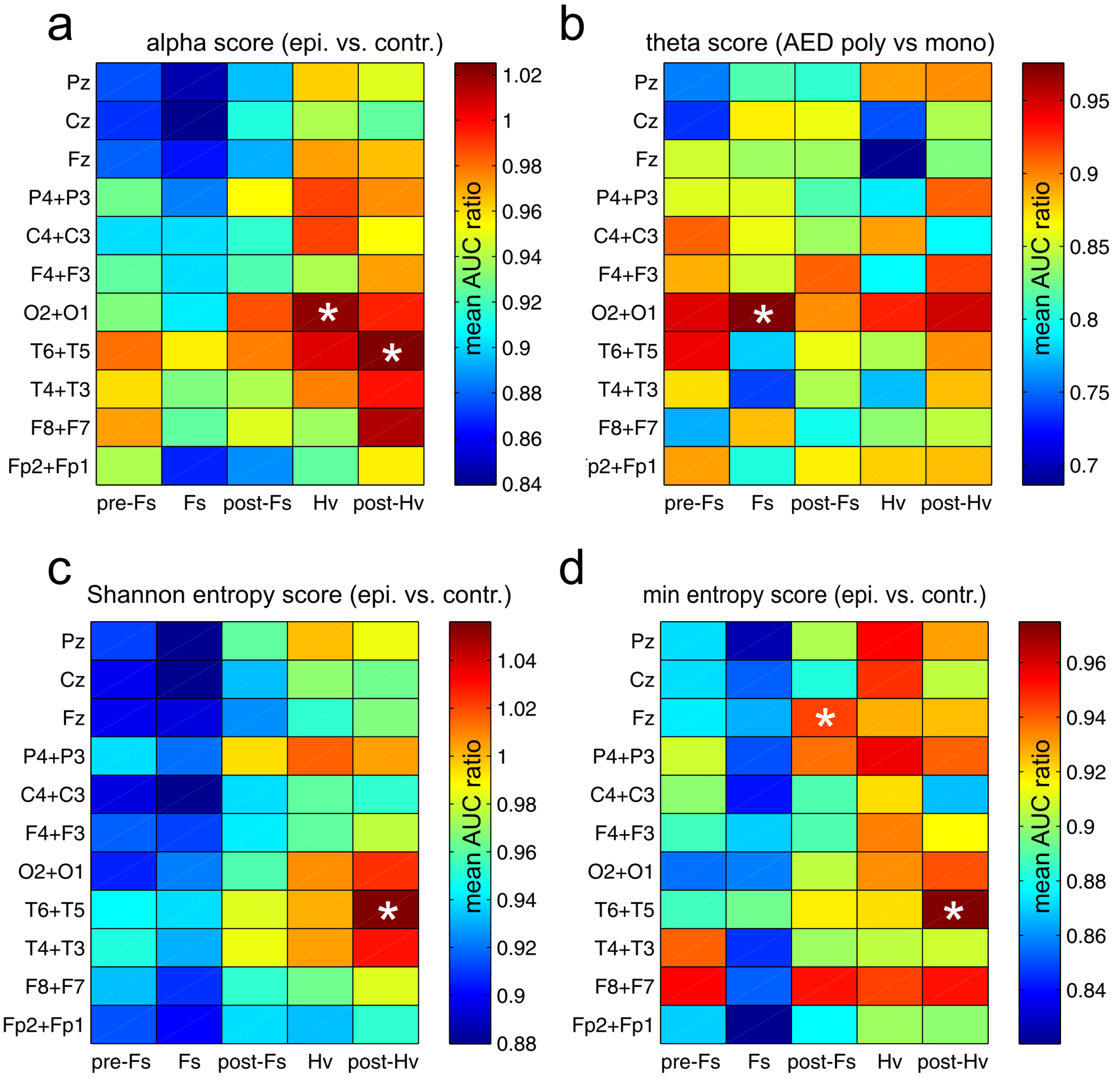

Trends in the optimization of alpha-, entropy- and theta-score based classifiers with respect to individual channel/segment combinations (second step of two-step fitting - see Results). The results are displayed as mean AUC ratios (see Results) and are directly related to Fig. 2a and b. Asterisks represent most common channel/segment combinations that maximized classifier performance (see also Table 2).

Supplementary Table 1

Subject no.	Sex	Age	Age (first seizure)	Diagnosis	Secondary generalization of seizures	AEDs taken by subject	EEG visual review	Comorbidities (ICD-10)
1	M	34	30	TLE	yes	LTG 200mg/d	normal	n/a
2	M	34	17	TLE	yes	CBZ 200mg/d	normal variant	n/a
3	M	56	14	TLE	yes	VPA 1300mg/d	normal	n/a
4	F	20	10	TLE	yes	VPA 600mg/d, TPM 400mg/d, LTG 300mg/d	non-specific path.	F39
5	F	50	n/a	TLE	yes	VPA 1500mg/d, CBZ 800mg/d, Clonazepam 3mg/d	normal	n/a
6	F	68	29	TLE	yes	clonazepam 1,5mg/d	IEDs	n/a
7	F	19	17	TLE	no	LEV 2000mg/d, LTG 300mg/d	normal variant	n/a
8	M	32	21	TLE	n/a	VPA 1800mg/d, LEV 1500mg/d	normal	n/a
9	F	37	1	TLE	yes	OXC 1200mg/d, TPM 400mg/d	IEDs	F48.9
10	F	39	34	TLE	yes	VPA 1600mg/d	normal variant	n/a
11	M	48	17	TLE	no	LEV 3000mg/d, VPA 2000mg/d, Clonazepam 6mg/d	normal variant	E78
12	F	46	17	TLE	yes	CBZ 1200mg/d, GBP 1800mg/d	non-specific path.	I83.9
13	M	50	17	TLE	yes	LTG 400mg/d, LEV 2250mg/d, TGB 30mg/d	IEDs	F48.9
14	F	53	22	TLE	yes	CBZ 1000mg/d, LTG 200mg/d, clonazepam 0.5mg/d	non-specific path.	F39, F06.7
15	F	53	16	TLE	yes	LTG 400mg/d, PHT 400mg/d, LEV 3000mg/d	IEDs	F33
16	F	39	6	TLE	no	CBZ 750mg/d	normal variant	I35, I33, st. post I63, st. post temporal res.
17	F	37	n/a	TLE	yes	LTG 400mg/d, CBZ 1800mg/d	non-specific path.	E78
18	F	52	1	TLE	yes	LEV 2000mg/d, GBP 2400mg/d	IEDs	I49.9
19	F	28	8	TLE	no	GBP 1800mg/d, TPM 300mg/d	IEDs	E66
20	F	27	22	FLE	yes	OXC 1200mg/d	IEDs	n/a
21	M	60	50	FLE	yes	-	non-specific path.	I25(o)
22	F	30	12	FLE	no	-	normal variant	E80.2
23	M	27	17	FLE	no	alprazolam 0,25mg/d	normal	n/a
24	F	26	12	FLE	yes	LTG 200mg/d, diazepam 5mg/d	normal	n/a
25	F	62	56	FLE	yes	CBZ 1000mg/d, LTG 100mg/d	normal variant	I10, E78, F41.2, M50, D25(o), N60(o)
26	F	50	48	FLE	yes	-	non-specific path.	E78

27	M	26	n/a	FLE	no	LEV 3500mg/d,<br>CBZ 1000mg/d,<br>TPM 100mg/d	non-specific path.	F44.5, G80
28	M	18	17	FLE	no	-	normal variant	n/a
29	M	24	7	FLE	no	CBZ 900mg/d,<br>TPM 200mg/d	norma	n/a
30	F	57	12	FLE	no	LEV 2500mg/d,<br>VPA 1600mg/d	normal	n/a
31	F	25	15	FLE	yes	OXC 1800mg/d	IEDs	n/a
32	F	25	7	FLE	yes	LEV 2000mg/d,<br>TPM 200mg/d	normal	F44.5
33	F	54	11	FLE	yes	OXC 1500mg/d,<br>LEV 2000mg/d	normal	I10, E78,<br>st. post partial parietal res.
34	F	34	5	FLE	yes	LEV 2000mg/d,<br>VPA300mg/d	IEDs	n/a
35	F	34	n/a	FLE	yes	CBZ 1200mg/d,<br>LEV 2500mg/d,<br>TPM 250mg/d	IEDs	n/a
36	F	35	15	FLE	yes	VPA 1000mg/d,<br>CBZ 800mg/d	IEDs	F61, J45
37	F	23	n/a	FLE	yes	VPA 2000mg/d,<br>CBZ 1500mg/d	IEDs	n/a
38	F	20	7	FLE	yes	VPA 1250mg/d,<br>GBP 900mg/d,<br>OXC	non-specific path.	F71, st po I63,<br>E03, I49.9
39	F	26	20	FLE	yes	VPA 1800mg/d	normal	n/a
40	M	40	2	FLE	yes	VPA 2000mg/d,<br>TPM 225mg/d	IEDs	E78, F70
41	F	37	33	FLE	yes	GBP 1200mg/d	normal variant	n/a
42	F	31	27	FLE	yes	CBZ 1200mg/d,<br>TPM 100mg/d	non-specific path.	n/a
43	F	23	0	FLE	no	CBZ 600mg/d,<br>VGB 1000mg/d	non-specific path.	B18,<br>st. post C92.0
44	F	22	15	FLE	yes	TPM 50mg/d,<br>LEV 3000mg/d,<br>LTG 175mg/d	IEDs	st. post corpus callosotomy,<br>st. post partial frontal resection
45	M	21	14	FLE	yes	LEV 4000mg/d,<br>VPA 1000mg/d,<br>TPM 200mg/d	normal variant	n/a
46	M	26	n/a	FLE	n/a	LTG 600mg/d,<br>LEV 3500mg/d	normal	n/a
47	F	35	10	FLE	yes	VPA 200mg/d,<br>LEV 2500mg/d,<br>GBP 2400mg/d	normal variant	n/a
48	F	31	14	FLE	no	VPA 2000mg/d,<br>LTG 250mg/d,<br>LEV 2250mg/d	IEDs	R55
49	M	27	10	FLE	no	LEV 2000mg/d	normal	I49.9
50	F	20	0	FLE	no	VPA 450mg/d,<br>STP 500mg/d,<br>CLB 20mg/d	normal variant	st. post VNS implantation
51	F	31	n/a	FLE	yes	TPM 250mg/d,<br>LTG 200mg/d,<br>VPA 600mg/d	non-specific path.	st. post G03,<br>G91(o), C71(o)
52	M	35	n/a	headache	n/a	LEV 1000mg/d	IEDs	H81, obs. quoad<br>G40 – negative
53	F	41	n/a	headache	n/a	-	non-specific path.	E34.9, F33
54	F	38	n/a	headache	n/a	VPA 1000mg/d	non-specific path.	E03, F31
55	M	47	n/a	headache	n/a	-	normal variant	n/a

56	F	44	n/a	headache	n/a	-	non-specific path.	I67.1, F43.2
57	F	43	n/a	headache	n/a	-	non-specific path.	G43
58	F	21	n/a	headache	n/a	-	normal	n/a
59	M	48	n/a	headache	n/a	-	non-specific path.	st. post G00
60	F	37	n/a	headache	n/a	-	normal variant	n/a
61	F	19	n/a	headache	n/a	-	normal variant	G44.2
62	F	39	n/a	headache	n/a	-	non-specific path.	G43
63	F	25	n/a	headache	n/a	-	normal	G44.2
64	F	55	n/a	headache	n/a	-	normal	G44.2
65	F	44	n/a	headache	n/a	-	normal	n/a
66	F	36	n/a	PNES	n/a	LEV 2750mg/d	IEDs	n/a
67	F	20	n/a	PNES	n/a	VPA 3000mg/d	IEDs	E03
68	F	21	n/a	PNES	n/a	VPA 600mg/d	normal	n/a
69	F	20	n/a	PNES	n/a	-	non-specific path.	n/a
70	F	45	n/a	PNES	n/a	OXC 1500mg/d	normal variant	n/a
71	F	24	n/a	PNES	n/a	CBZ 900mg/d	non-specific path.	F11
72	M	31	n/a	PNES	n/a	VPA 300mg/d	normal variant	F61
73	F	19	n/a	PNES	n/a	LTG 100mg/d	normal	n/a
74	F	26	n/a	PNES	n/a	LTG 200mg/d	normal	n/a
75	F	57	n/a	PNES	n/a	LTG	n/a	n/a
76	M	20	n/a	PNES	n/a	-	normal	n/a
77	F	20	n/a	PNES	n/a	VPA 300mg/d, CBZ 300mg/d	non-specific path.	n/a
78	F	36	n/a	PNES	n/a	OXC 300mg/d	non-specific path.	n/a

Detailed clinical data of the studied patient group. Abbreviations: FLE - frontal lobe pilepsy, TLE - temporal lobe epilepsy, PNES - psychogenic nonepileptic seizures, VNS - vagus nerve stimmulation, IEDs - interictal discharges. Where clinical data were not availible (or not applicable) respective fields are marked with n/a. Antiepileptic drugs are denoted by standard abbreviations (drug dosing is not specified if it was not avalibe), comorbidities are represented by ICD-10 codes.
